# A discovery and verification approach to pharmacovigilance using electronic healthcare data

**DOI:** 10.3389/fphar.2024.1426323

**Published:** 2024-09-04

**Authors:** Louis Dijkstra, Tania Schink, Roland Linder, Markus Schwaninger, Iris Pigeot, Marvin N. Wright, Ronja Foraita

**Affiliations:** ^1^ Leibniz Institute for Prevention Research and Epidemiology – BIPS, Bremen, Germany; ^2^ Techniker Krankenkasse – TK, Hamburg, Germany; ^3^ Institute for Experimental and Clinical Pharmacology and Toxicology, University of Lübeck, Lübeck, Germany; ^4^ Faculty of Mathematics and Computer Science, University of Bremen, Bremen, Germany; ^5^ Section of Biostatistics, Department of Public Health, University of Copenhagen, Copenhagen, Denmark

**Keywords:** Borda count, DOAC, drug safety, GEPARD, machine learning, medical records

## Abstract

**Introduction:**

Pharmacovigilance is vital for drug safety. The process typically involves two key steps: initial signal generation from spontaneous reporting systems (SRSs) and subsequent expert review to assess the signals’ (potential) causality and decide on the appropriate action.

**Methods:**

We propose a novel discovery and verification approach to pharmacovigilance based on electronic healthcare data. We enhance the signal detection phase by introducing an ensemble of methods which generated signals are combined using Borda count ranking; a method designed to emphasize consensus. Ensemble methods tend to perform better when data is noisy and leverage the strengths of individual classifiers, while trying to mitigate some of their limitations. Additionally, we offer the committee of medical experts with the option to perform an in-depth investigation of selected signals through tailored pharmacoepidemiological studies to evaluate their plausibility or spuriousness. To illustrate our approach, we utilize data from the German Pharmacoepidemiological Research Database, focusing on drug reactions to the direct oral anticoagulant rivaroxaban.

**Results:**

In this example, the ensemble method is built upon the Bayesian confidence propagation neural network, longitudinal Gamma Poisson shrinker, penalized regression and random forests. We also conduct a pharmacoepidemiological verification study in the form of a nested active comparator case-control study, involving patients diagnosed with atrial fibrillation who initiated anticoagulant treatment between 2011 and 2017.

**Discussion:**

The case study reveals our ability to detect known adverse drug reactions and discover new signals. Importantly, the ensemble method is computationally efficient. Hasty false conclusions can be avoided by a verification study, which is, however, time-consuming to carry out. We provide an online tool for easy application: https://borda.bips.eu.

## 1 Introduction

**FIGURE 1:**
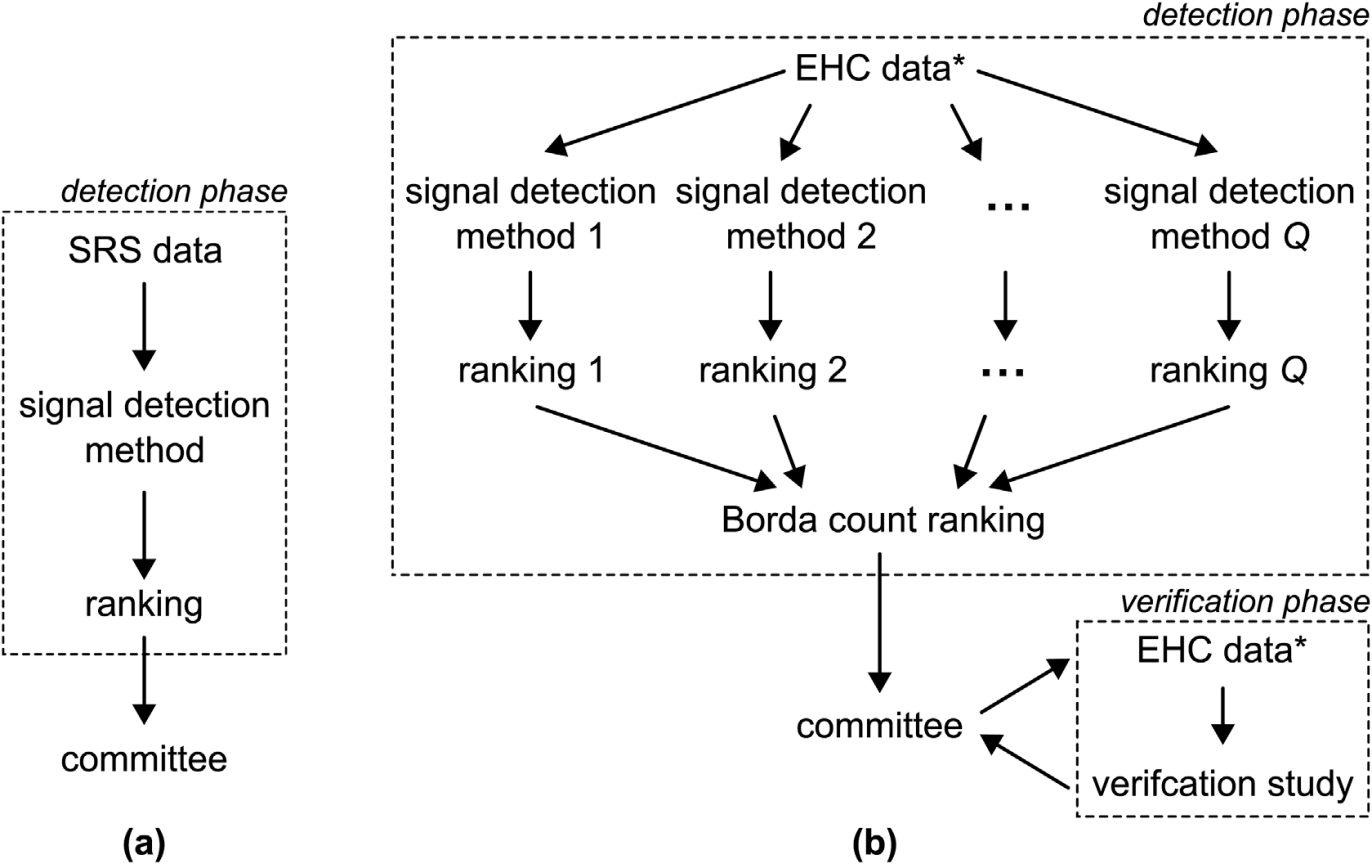
**(A)** The conventional pharmacovigilance approach involves applying a single detection method to SRS data to generate a single ranking of ADRs pairs. This ranking is subsequently submitted to a committee of medical experts. **(B)** The approach proposed in this paper entails the application of Q distinct signal detection methods to EHC data, resulting in Q rankings. These rankings are then amalgamated into a unified ranking using the Borda count method. The consolidated ranking is then presented to the committee. Furthermore, the option to conduct a verification study is provided to the committee as well. The * indicates that, preferably, the EHC data are split into two sets: one for detection and one for the verification phase.

Evidence of the safety of newly approved drugs is often limited. The pivotal randomized clinical trials (RCTs) are powered to assess efficacy so that the sample sizes are too small to examine rare safety outcomes. Patients in RCTs usually have to fulfill several inclusion criteria and especially vulnerable groups such as pregnant women, elderly, and multi-morbid persons are often excluded or underrepresented. Moreover, patients are followed up very closely under controlled conditions over a limited period of time. Post-market surveillance or pharmacovigilance is, therefore, essential to guarantee the safety of drugs in routine care (Routledge, 1998; World Health Organization, 2002; Härmark and van, 2008; Suling and Pigeot, 2012; Aronson, 2023). Its primary goal is to promptly identify any previously unknown adverse drug reactions (ADRs) associated with drugs already on the market, allowing necessary precautions to be taken to safeguard the population. The post-market surveillance process typically comprises of three key elements or stages, as illustrated in Figure 1A: 1) the use of spontaneous reporting system (SRS) data, 2) a signal detection phase, and 3) the evaluation of the results by a committee of medical experts. In this context, we introduce a novel discovery and verification approach to pharmacovigilance, shown in Figure 1B. We start by considering each of the three components of the conventional approach individually and then propose potential improvements for each step.

Pharmacovigilance traditionally relies on SRS data as the primary data source (Routledge, 1998; World Health Organization, 2002; Suling and Pigeot, 2012; Huang et al., 2014; Bailey et al., 2016; Alomar et al., 2020; Noguchi et al., 2021; Costa et al., 2023; Cutroneo et al., 2024). In the last decades, research has shifted its focus to electronic healthcare (EHC) data which encompass individual patients’ drug prescriptions and medical events over time, in addition to personal details, such as age, sex, region of residence and other factors (Suling and Pigeot, 2012; Coloma et al., 2012; Zorych et al., 2011; Patadia et al., 2015; Li et al., 2015; Pacurariu et al., 2015). EHC data offer several advantages over SRSs: 1) the total count of patients prescribed a drug and/or experiencing an ADR is available, whereas spontaneous reports are only filed when the drug was prescribed and an ADR occurred; 2) issues of under- and over-reporting are less prominent (Schroeder, 1998; van der Heijden et al., 2002); and 3) some EHC databases include follow-up data for several million individuals (Haug and Schink, 2021). There is a strong push to make electronic healthcare data more accessible. One such initiative is the European Health Data Space (EHDS; (European Commission, 2024)), which aims to standardize data formats, enhance interoperability, and ensure privacy protections across the European Union.

A wide variety of statistical signal detection methods have been proposed for the detection phase in which one aims to identify associations between drugs and ADRs, often referred to as signals (Pacurariu et al., 2015; Simpson, 2011; Arnaud et al., 2017; Dijkstra et al., 2020). These methods encompass a wide spectrum, including disproportionality measures (e.g., reporting odds ratio (Stricker and Tijssen, 1992)), hypothesis tests (e.g., Poisson test (DuMouchel, 1999)), Bayesian shrinkage estimates (e.g., Bayesian confidence propagation neural network or BCPNN (Bate et al., 1998; Norén et al., 2006)), random forests (RFs (Breiman, 2001)), and sparse regression methods like LASSO (Caster et al., 2010; Tibshirani, 1996). Each method assigns a score to each of the drug-ADR pairs in the data. Following signal generation, these scores are utilized to create a ranking of the drug-ADR pairs, reflecting the strength of associations between them.

Each of these signal detection methods possesses distinct strengths and weaknesses. For instance, the widely-used BCPNN excels in cases involving innocent bystanders, in which a drug is mistakenly linked to an ADR since it is frequently co-prescribed with the actual causative drug (Dijkstra et al., 2020). However, it has the limitation of evaluating each drug-ADR pair independently. In contrast, LASSO, while less effective in scenarios with innocent bystanders (Dijkstra et al., 2020), does not have this constraint and can assess multiple ADRs simultaneously, even accommodating potential confounders (Dijkstra et al., 2020; Caster et al., 2010; Tibshirani, 1996). RFs stand out due to their capability to handle non-linear relationships (Breiman, 2001); a feature that sets them firmly apart from the other methods. The choice of signal detection method is, therefore, non-trivial and depends on many factors, as highlighted in previous research (Dijkstra et al., 2020; Candore et al., 2015). In such scenarios, ensemble methods have shown their effectiveness. They have the capacity to combine the strengths of individual classifiers and mitigate some of their limitations (Rokach, 2009; Antczak, 2016; Tabassum and Ahmed, 2016). Furthermore, ensemble methods are particularly adept at handling noisy data (Ho et al., 1994).

After the signal detection phase, the resulting ranking of drug-ADR pairs is presented to a committee of medical experts, which triages the signals and decides on any possible actions, e.g., issue warnings, request label changes or, in extreme cases, recommend to withdraw the license (Alomar et al., 2019).

We propose the alternative approach to pharmacovigilance shown in Figure 1B, with the goal of addressing the challenges outlined earlier. We choose to utilize EHC data which not only offers the previously mentioned advantages but also provides the possibility to execute a subsequent verification phase, as elaborated on later. Second, to leverage the strengths and address the limitations of various signal detection methods, we advocate for employing an ensemble of more than one method, see Figure 1B. The scores generated by these methods result in varying rankings, which are then aggregated into a unified ranking using the Borda count, a method originally developed in the field of electoral mathematics (Emerson, 2011). The Borda count ranking is advantageous as it emphasizes consensus (Saari, 1985; 2022). Furthermore, we extend the options available to the committee by introducing the option to conduct a verification study using pharmacoepidemiological methods. This verification phase allows the committee to assess whether signals persist when standard methods, such as controlling for confounding, are applied.

In conclusion, we define a new approach to pharmacovigilance that utilizes an ensemble of signal detection methods and offers the committee of medical experts the opportunity to verify select signals through a tailored pharmacoepidemiological study. We assess the approach’s applicability by conducting a case study using real-world EHC data, where we apply an ensemble of four established signal detection methods to uncover potential unknown ADRs associated with the direct oral anticoagulant rivaroxaban. We perform a pharmacoepidemiological signal verification study on a subset of interesting signals selected by the committee.

## 2 Methods

This section outlines the proposed discovery and verification approach in general terms, structured around three phases as shown in Figure 1B: 1) signal detection, 2) signal triage, and 3) signal verification. To prevent any potential confusion, we define certain terms used throughout the paper here, as their interpretations may vary across different fields. Here, we define an adverse drug reaction (ADR) as any individual event that can be reported to a SRS or documented in electronic healthcare records, even if the event is not commonly thought of as a reaction to a drug. A drug-ADR pair denotes a combination of a specific drug and a particular ADR. A signal is a drug-ADR pair that is deemed associated after applying a signal detection method. The identification of a signal is contingent upon the chosen detection method and the threshold used to determine whether the drug-ADR pair should be submitted to the committee. Additionally, we use the term health outcome to denote a collection of various drug-ADR pairs, typically defined by the committee of medical experts.

### 2.1 Signal detection

Typically, the detection of potential previously-unknown ADRs is carried out using a single signal detection method. This method assigns a score to each drug-ADR pair within the dataset, reflecting the strength of the association between the drug and the ADR in question. These scores may take various forms, such as odds ratios (van Puijenbroek et al., 2002), p -values (DuMouchel, 1999), or penalized regression coefficients (Caster et al., 2010). Subsequently, these scores are utilized to create a ranking of the drug-ADR pairs. This ranking is then presented to a committee of medical experts for discussion and signal triage (see Figure 1A). We use the convention throughout that the lowest rank of 1 corresponds to the strongest signal and that the highest rank corresponds to no signal.

We propose the use of an ensemble of more than one signal detection methods. Given the diverse nature of the scores produced by signal detection methods, it is challenging to directly aggregating them into a single score. Instead, we opt for aggregating the rankings generated by the various signal detection methods into a unified ranking using the Borda count method (Emerson, 2011; Saari, 2022; de Borda, 1781). Drawing inspiration from electoral mathematics, the Borda count treats each signal detection method as a “voter” that ranks drug-ADR pairs in order of preference. The Borda count for each drug-ADR pair is calculated as the sum of the individual ranks assigned by the voters/methods. The drug-ADR pair with the lowest Borda count is deemed to exhibit the strongest signal and is positioned at the top, while the pair with the highest Borda count is placed at the bottom, indicating no signal. Tournament-style counting is employed to handle ties (Narodytska and Walsh, 2014). See the Supplementary Material for a formal definition of Borda count ranking.

The essence of the Borda count ranking lies in capturing the “consensus” among the voters/methods, rather than relying on a simple majority (Emerson, 2011; de Borda, 1781). To see this, let us consider an example where the ensemble consists of three methods and the EHC dataset contains five drug-ADR pairs: A, B, C, D and E. Table 1 shows the ranking of each method. For instance, Method 1 assigned D rank 1, corresponding to the strongest signal, B second etc., and ending with A which is deemed to weakest signal. The right column shows the ranking using the Borda method, with the corresponding Borda count shown in brackets. For example, B is ranked second by all methods, resulting in a Borda count of 6. D ranked first by Method 1 and Method 2, but last by Method 3, giving it a Borda count of 1+1+5=7 . The remaining drug-ADR pairs are ranked similarly. In this context, if relying solely on Method 1 or 2, D would be considered the strongest signal, but Method 3 ranks it last. Conversely, drug-ADR pair B consistently receives a high rank from all methods, making it more likely to be a signal of interest. This illustrates the consensus-seeking property of the Borda count method.

**TABLE 1:** Example of Borda count ranking with three signal detection methods and five drug-ADR pairs (A, B, C, D,and E).

Rank	Method 1	Method 2	Method 3	Borda ranking (count)
1	D	D	A	B (6)
2	B	B	B	D (7)
3	C	A	C	A (9)
4	E	C	E	C (10)
5	A	E	D	E (13)

### 2.2 Signal triage

Following the signal detection phase, the committee of medical experts is provided with the drug-ADR pairs ranking derived from the Borda count. This committee is entrusted with the task of filtering out signals, aiming to identify severe and previously unknown ADRs and taking appropriate actions.

It is important to note that expert knowledge in this part of the process is paramount. Subject-matter knowledge is required to assess the biological plausibility that an ADR is indeed caused by the drug currently under suspicion. Relying solely on the top-ranked drug-ADR pairs proves insufficient, as these are likely to encompass known ADRs as well, whereas previously unknown ADRs are more likely to be further down the list, i.e., they tend to generate weaker signals. The possibility of the drug in question to be an innocent bystander (Dijkstra et al., 2020), or whether the signal is due to indication bias, has to be ruled out.

Based on medical or pharmacological considerations, the committee has the option to merge specific ADRs. This involves aggregating ICD codes from multiple signals into a single health outcome. See Section 3.3 for an example. A formal definition of a health outcome can be found in the Supplementary Material.

To facilitate the signal triage process, we have developed an online tool based on Borda count rankings, accessible at https://borda.bips.eu. Our R-Shiny app computes the Borda count ranking using uploaded data and offers graphical visualizations to aid the selection of drug-ADR pairs for further investigation.

### 2.3 Signal verification

The presented approach includes a verification phase (see Figure 1B), providing the committee with the opportunity to assess the reliability of filtered signals through an observational study focused on the specific drug-ADR pair or health outcome of interest. This study should be designed to minimize bias and control for potential confounding, for instance, by employing a new user active comparator approach (Lund et al., 2015), evaluating all potential confounders, and utilizing appropriate statistical methods. It is important to note that due to the substantial number of drug-ADR pairs, systematically verifying all signals through traditional pharmacoepidemiological studies is not feasible. As a result, verification is limited to a small subset of drug-ADR pairs/health outcomes.

## 3 Case study

We demonstrate the applicability of the approach outlined in the previous section using a case study. We begin by introducing the data source and the drug of interest, rivaroxaban (RVX, ATC: B01AF01). We are focusing on this drug in particular because it is central to the PV-Monitor project from which this work stems. For further details, please refer to the Funding section. The subsequent part of the section adheres to the same structure as the preceding one, starting with signal detection, followed by signal triage, and concluding with signal verification. The results of the case study are available at https://borda.bips.eu as well. The website also provides the option to conduct a similar analysis with one’s own dataset.

### 3.1 Data source and exposure of interest

We used the German Pharmacoepidemiological Research Database (GePaRD) that is based on claims data from four statutory health insurance (SHI) providers in Germany. It currently includes information on approximately 25 million persons who have been insured with one of the participating providers since 2004 or later (Haug and Schink, 2021).

To illustrate our methodology, we focus on a single drug: RVX, a direct oral anticoagulant (DOAC) that has been approved for, e.g., the prevention of stroke and systemic embolism in patients with atrial fibrillation, the treatment of deep vein thrombosis and pulmonary embolism, and prevention of recurrent deep vein thrombosis and pulmonary embolism in adult patients, and for the prevention of venous thromboembolism in adult patients undergoing elective hip or knee replacement surgery. As is the case with other anticoagulants, clinical studies of rivaroxaban identified hemorrhage as an important safety outcome (Ruff et al., 2014). Since the approval of RVX on the German market in 2008, numerous ADRs have been reported to spontaneous reporting systems (Morgovan et al., 2023). In this case study, however, we are interested in as of yet unknown ADRs. GePaRD and the cohort of the signal detection study are described in detail in the Supplementary Material.

### 3.2 Signal detection

#### 3.2.1 General principle

We employ four signal detection methods as the foundation for the ensemble. These methods consist of the BCPNN (Norén et al., 2006)), the LGPS (Schuemie, 2011), LASSO (Caster et al., 2010; Tibshirani, 1996), and RFs (Breiman, 2001). See the Supplementary Material for a full description of all four methods. We opt for the BCPNN method, since it is the method of choice for SRSs. The LGPS is a disproportionality method tailored specifically to longitudinal data, demonstrating superior performance in the Observational Medical Outcomes Partnership (OMOP) competition (Schuemie, 2011; The Observational Medical Outcomes Partnership, 2010). The inclusion of LASSO is motivated by its ability to regress all ADRs against the drug of interest while considering patient information and accounting for potential confounding variables. RFs are included in our analysis since they can handle non-linear effects (Breiman, 2001). We use the corrected version of the impurity measure for the RFs (Nembrini et al., 2018). It is important to emphasize that this serves as an illustrative example, and alternative signal detection methods could be employed as well.

We included persons in the signal detection study who.

1. Had been insured with the health insurance Techniker Krankenkasse (TK), which is one of the largest nationwide SHI providers in Germany, for at least 12 consecutive months between January 2015 and December 2016;
2. Who had at least one dispensation of RVX at least 90 days after cohort entry and before cohort exit, respectively, and
3. Who had no DOAC use within 12 months preceding cohort entry.

In our analysis, we incorporate covariates such as age, sex, other diagnoses, and prescribed medications to address potential interactions. See the Supplementary Material for details.

#### 3.2.2 Results

**FIGURE 2:**
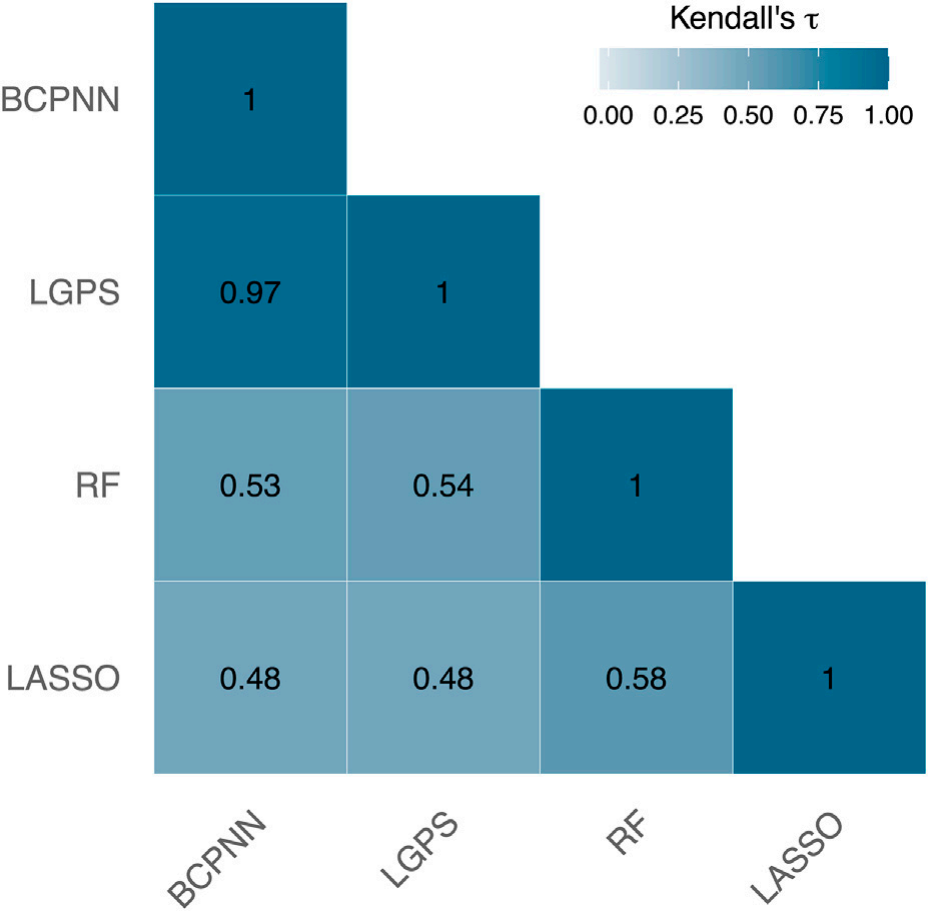
Kendall’s τ correlations between the rankings of the BCPNN, LGPS, RF and LASSO. The correlation values fall within the range of [−1,1] . However, for clarity, the legend only depicts the positive range [0,1].

The dataset included 3,795 ICD-codes that were treated as potential ADRs. Figure 2 illustrates the Kendall’s τ correlations among the rankings produced by the four signal detection methods forming the basis of the ensemble method, i.e., BPCNN, LGPS, LASSO, and RF. The disproportionality methods, BCPNN and LGPS, lead to similar rankings. The LASSO and the RF, however, differ substantially from the others.

For instance, consider the ADR acute cystitis with the ICD-code N30.0, which is ranked quite disparately by these methods. BCPNN, LGPS, and RF position this ICD-code among the top 2% of the strongest signals, whereas LASSO does not even include it in the top 10%. Depending solely on the LASSO output could result in overlooking this ADR as a potential signal.

Table 2 presents the top 10 ICD-codes with the highest rankings according to the Borda count. It is worth noting that K92.2 (gastrointestinal hemorrhage, unspecified) is a recognized ADR associated with DOACs.

**TABLE 2:** Top ten ICD-10-GM codes for rivaroxaban according to the Borda ranking.

Rank	ICD-10 code	Description
1	D50.8	Other iron deficiency anemias
2	J69.0	Pneumonitis due to inhalation of food and vomit
3	K92.2	Gastrointestinal hemorrhage (unspecified)
4	K31.8	Other specified diseases of stomach and duodenum
5	D50.0	Iron deficiency anemia secondary to blood loss (chronic)
6	C20	Malignant neoplasm of rectum
7	K25.0	Acute gastric ulcer with hemorrhage
8	E86	Volume depletion
9	I27.2	Other secondary pulmonary hypertension
10	N17.9	Acute kidney failure (unspecified)

### 3.3 Signal triage

#### 3.3.1 General principle

A committee of pharmacoepidemiologists, pharmacologists, physicians and statisticians reviewed the rankings and discussed plausibility, severity and the novelty of the signals.

#### 3.3.2 Results

Based on the ranking obtained during the signal detection phase, the committee identified four health outcomes of interest: acute liver injury (ALI), acute cystitis (CYS), epilepsy and seizures (EPI), and sepsis. Additionally, two health outcomes were chosen as positive controls – gastrointestinal bleeding (GB) and intracranial bleeding (ICB) – as they are known adverse outcomes of RVX. See the Supplementary Material for the ICD codes corresponding to each health outcome.

**FIGURE 3:**
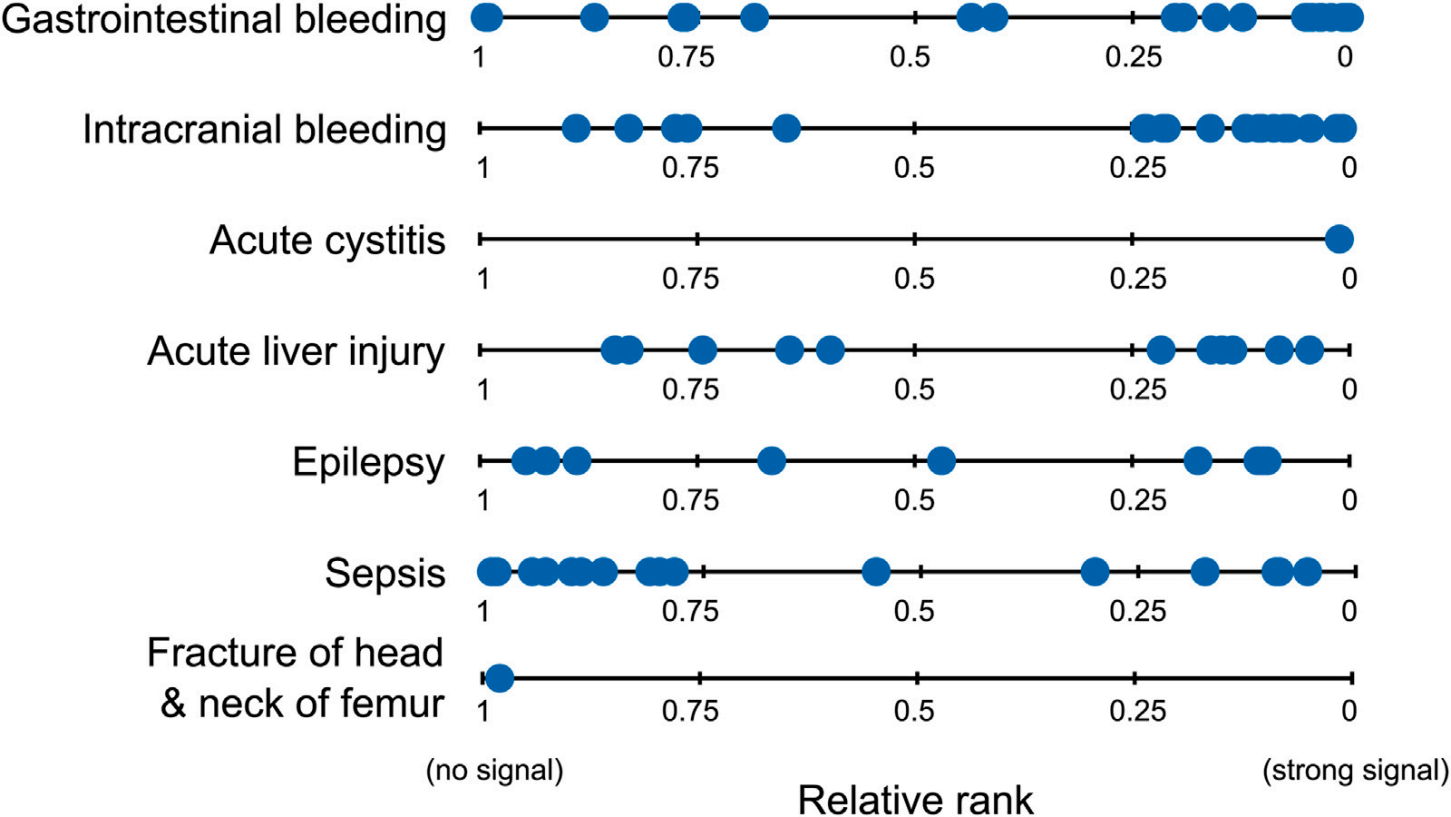
The relative ranks represent the positions of seven adverse outcomes, with five consisting of multiple individual ADRs. Each dot represents an individual ICD-code. A relative rank of 1 signifies the highest rank, indicating no signal, while a relative rank of 0 indicates the top rank, suggesting a very strong signal.

This selection was made based on medical plausibility, whether the event was already known before, and with the help of plots as shown in Figure 3. This figure shows the relative Borda ranks for the ICD-codes associated with each of the six health outcomes and a negative control: Fracture of head and neck of femur (S72.0). Each dot represents an individual ICD-code. A relative rank of 1 indicates the ADR is ranked last (indicating no signal), while a relative rank of 0 signifies the item is at the top of the ranking (indicating a strong signal). We prefer to use the relative rank for its clarity.

GB and ICB are established adverse outcomes of RVX, with many associated ADRs exhibiting strong signals. Acute cystitis, corresponding to a single ADR (N30.0), produces a very strong signal. The ADRs linked to epilepsy and sepsis exhibit significant variation; there are both groups of ADRs that display strong associations and those that suggest there is no connection with RVX. Especially the latter two can benefit from performing a verification study. It is evident from the bottom row that there is no signal for the negative control. The negative control is, therefore, not considered in the verification study.

### 3.4 Signal verification

#### 3.4.1 General principle

The verification study was designed as an active comparator case-control study nested in a cohort of new users of RVX and phenprocoumon (PPC, ATC: B01AA04, the most frequently used vitamin K antagonist in Germany) with atrial fibrillation and included data from 2011 to 2017. Cases were defined as patients with a diagnosis of the respective outcome of interest. Each case was matched with up to 10 controls by sex, age at index day ( ±1 year) and SHI using risk set sampling (Goldstein and Langholz, 1996) with time in cohort as the time axis to ensure a similar follow-up as for the corresponding case. Eligible patients hospitalized for any reason at the index date of the case were excluded from the set of potential controls. Cases were eligible for selection as controls before their index date, and controls could be selected more than once (Rothman et al., 2008). The outcome of interest was defined based on the signals identified by the expert committee. A list of potentially confounding variables was established to each health outcome (see Supplementary Material for details). Conditional logistic regression was used to estimate crude and confounder-adjusted odds ratios (ORs) with 95% confidence intervals (95% CI). We report ORs that compare the current use of RVX with PPC (active comparator) and to no exposure to RVX or PPC at index day. However, the in- and exclusion criteria for the signal detection step and the verification study differ. The verification study contains only individuals with atrial fibrillation whereas the signal detection study contains all insurants that were exposed to RVX.

#### 3.4.2 Results

The study cohort of the verification study was based on 97,400 new users of PPC and 71,917 users of RVX. The median age at cohort entry was 73 (IQR: 65, 79) for RVX and 75 (IQR: 69, 81) for PPC new users. The proportion of women was 47% (RVX) and 48% (PPC). We observed 5,053 cases of acute cystitis, 322 cases of acute liver injury, 3,070 cases of epilepsy, 3,504 cases of sepsis, 6,705 cases of gastrointestinal bleeding, and 2,974 cases of intracranial bleeding. A full description of the study cohort, including baseline characteristics, relevant medical history and dispensed medication at cohort entry, can be found in the Supplementary Material.

Table 3 shows that the adjusted risk of gastrointestinal bleeding was increased in current single use of RVX compared to current single use of PPC (OR = 1.37, 95% CI: (1.27; 1.47)) but decreased for intracranial bleeding (OR = 0.74, 95% CI: (0.66; 0.83)). This is what we expected from the literature (Hohnloser et al., 2017). The risk of cystitis was slightly increased (OR = 1.11, 95% CI: (1.02; 1.21)) whereas the risk of acute liver injury was much lower in patients using RVX compared to PPC (OR = 0.50, 95% CI: (0.35; 0.72)). Patients receiving current treatment with RVX showed a considerably higher risk of epilepsy and seizures compared to PPC users (OR = 1.26, 95% CI: (1.11; 1.43)). The risk of sepsis was similar in current users of RVX compared to current use of PPC (OR = 1.10, 95% CI: (0.99; 1.23)).

**TABLE 3:** Results of the verification study: matched crude and adjusted odds ratios with corresponding 95% confidence intervals.

			Current use of RVX at index day		Current use of RVX at index day vs. current single use of PPC at index day	
Health outcome	Cases N	Controls N	in Cases N (%)	in Controls N (%)	Crude OR (95% CI)	Adjusted^b^ OR (95% CI)
Gastrointestinal bleeding^a^	6,705	67,019	1,635 (24.4%)	13,817 (20.6%)	1.22 (1.14; 1.31)	1.37 (1.27; 1.47)
Intracranial bleeding^a^	2,974	29,720	515 (17.3%)	5,959 (20.1%)	0.72 (0.64; 0.80)	0.74 (0.66; 0.83)
Acute cystitis	5,053	50,424	1,123 (22.2%)	10,590 (21.0%)	1.09 (1.00; 1.19)	1.11 (1.02; 1.21)
Acute liver injury	322	3,220	52 (16.1%)	688 (21.4%)	0.50 (0.35; 0.71)	0.50 (0.35; 0.72)
Epilepsy	3,070	30,676	617 (20.1%)	6,362 (20.7%)	1.34 (1.19; 1.51)	1.26 (1.11; 1.43)
Sepsis	3,504	35,022	670 (19.1%)	7,231 (20.6%)	1.01 (0.91; 1.12)	1.10 (0.99; 1.23)

RVX, rivaroxaban. PPC, phenprocoumon. OR, odds ratio.

^a^ Positive control.

^b^ Adjusted for outcome-specific comorbidities and co-medications (see Supplementary Material for details).

## 4 Discussion and conclusion

In this paper, we introduced a novel approach to pharmacovigilance. Conventionally, a single signal detection method is applied to SRS data, and the resulting ranking of drug-ADR pairs is presented to a committee of medical experts. Here, we enhance this process in three significant ways: First, we opt for EHC data due to its richness and its capacity to facilitate a pharmacoepidemiological study; something that cannot be accomplished with SRS data. Second, we simultaneously apply multiple signal detection methods, aggregating the obtained rankings through the Borda count and we provide the committee with a visual representation for defining health outcomes. See, for example, Figure 3. Third, we recommend verifying a selection of health outcomes in a pharmacoepidemiological verification study. Such a study can be immensely beneficial, as it may offer support for either confirming the existence of the association or revealing its spurious nature (Platzbecker et al., 2023).

To illustrate this approach, we conducted a case study with the direct oral anticoagulant rivaroxaban (RVX) based on the healthcare claims database GePaRD. In our case study, the ensemble method comprised of four signal detection methods: BCPNN, LGPS, LASSO, and RF. After applying the ensemble method to the data, the resulting ranking was sent to the committee. During signal triage, a total of four health outcomes were selected to be further investigated in a pharmacoepidemiogical verification study: acute liver injury (ALI), acute cystitis (CYS), epilepsy and seizures (EPI), and sepsis. In addition, gastrointestinal bleeding (GB) and intracranial bleeding (ICB) served as positive controls, being recognized adverse health outcomes of RVX. We performed an active comparator, case-control study nested in GePaRD in which we compared patients with atrial fibrillation who initiated treatment with RVX or phenprocomoun (PPC). For each health outcome, possible confounding variables were collected from the literature and adjusted for. Our approach successfully identified both the positive controls GB and ICB. Additionally, the results indicated no significant safety issues in RVX users compared to PPC concerning the occurrences of ALI, CYS, and sepsis, which were ranked in the top third of the Borda count ranking, as depicted in Figure 3. However, the verification study confirmed an elevated risk for epilepsy and seizures in RVX users compared to PPC users. This finding was further investigated and published by Platzbecker et al. (2023). Within this paper, also the limitations of the pharmacoepidemiological studies are discussed in more detail. These examples illustrate that signal detection using EHC data is feasible, but also underscore the importance of verification to address biases such as confounding, selection, or measurement bias (Prada-Ramallal et al., 2019).

The ensemble method proposed for the signal detection phase is characterized by its flexibility, allowing for easy modification of the signal detection methods incorporated in the ensemble, and it is computationally fast. By employing an ensemble, the goal is to leverage the individual advantages of different methods while potentially mitigating some of their limitations (Rokach, 2009; Antczak, 2016; Tabassum and Ahmed, 2016). Moreover, ensemble techniques are adept at handling noisy data (Ho et al., 1994). The use of Borda count ranking for aggregation offers the advantage of independence from the interpretation or characteristics of the scores generated by each individual signal detection method. The method aims to promote consensus (Emerson, 2011; Saari, 2022), as discussed in Section 2. The case study highlights that individual signal detection methods may produce significantly different rankings, as shown in Figure 2. The Borda method also allows for assigning weights to the rankings produced by individual signal detection methods. This adaptability facilitates the prioritization of output from specific methods over others, proving particularly valuable when there is an expectation that a particular method will excel in the given scenario.

Conducting a pharmacoepidemiological verification study is generally laborious and if the study confirms a safety issue, a follow-up with sensitivity analyses to investigate different sources of potential bias is required. Hence, we considered only a limited number of health outcomes in our case study. It is important to recognize that the decision for the additional verification step in the presented approach leads to a time-consuming process. While this may be acceptable in certain situations, the committee tasked with evaluating signals must factor in this aspect when deciding on the course of action.

In the case study, we focused on a single medication rather than multiple drugs simultaneously, which is more typical in pharmacovigilance. However, this does not prohibit the application of the proposed approach to scenarios involving multiple drugs. The resulting Borda ranking presented to the committee would be longer; however, this is standard in the pharmacovigilance context. Most signal detection methods are computationally efficient and capable of handling large datasets with numerous drug-ADR pairs. Consequently, running multiple methods simultaneously should not present significant challenges.

It is standard practice to partition the dataset into two segments for detection and verification to avoid overfitting. Nevertheless, when the available data are limited, such as when the drug of interest is seldom prescribed or the ADR is rare, this approach might significantly diminish the power to detect relevant signals, possibly leaving crucial associations between drugs and ADRs unnoticed. In such scenarios, the option of not splitting the data could be considered; however, we caution that this decision should be taken into account when interpreting the results of the verification study. In our RVX case study, the datasets used for signal detection and the verification study overlap slightly (2 years of data from one health insurance company compared to data over 7 years from four health insurance companies). In addition, the inclusion and exclusion criteria for signal generation and the verification studies were very different. For more details, please see Section 3.2 and Section 3.4. In Platzbecker et al. (2023), we conducted a sensitivity analysis by repeating our main analysis without the data used for signal detection and were able to confirm our results.

The availability of EHC data is often delayed, meaning that there can be a significant gap between the occurrence of the event itself and the moment the observation becomes available for analysis. This delay must be taken into account when utilizing EHC data for detection, as it can be challenging, if not impossible, to detect ADRs for drugs that very recently entered the market. In those cases, SRS data might be a more appropriate choice, since reports tend to be processed quickly. However, for drugs that have been on the market for a longer time, EHC data may be preferable due to its richer nature, allowing for easier detection of rare ADRs and long-term effects. Therefore, we recommend users to carefully consider which data source to use for signal detection, as it heavily depends on their specific goals.

In efforts to improve post-market surveillance, research typically focuses on one or more of three key aspects: 1) the data source being used, 2) the statistical methods employed for signal detection, and 3) the signal triage process. Alongside traditional data sources such as SRSs or EHC data, alternative sources like social media posts, journal publications, sensor data, and reporting apps are also being explored (Lavertu et al., 2021; Satwika et al., 2021; Shin et al., 2022; Pilipiec et al., 2022). Instead of relying solely on a single source, some studies delve into multimodal approaches, integrating multiple sources simultaneously (Dimitriadis et al., 2023). In this particular study, our choice for the signal detection phase was EHC data since it can be used for the verification study as well. However, other data sources could also be used. However, alternative data forms such as SRS and social media are unsuitable for performing a pharmacoepidemiological verification study.

A considerable amount of research is directed towards enhancing the signal detection phase through the development of novel methodologies or the refinement of existing ones. Particularly in recent years, there has been a strong emphasis on artificial intelligence methods within the field (Bate and Hobbiger, 2021; Huysentruyt et al., 2021; Ball and Dal Pan, 2022; Kompa et al., 2022; Painter et al., 2023), although classical methods continue to be considered as well. For example, Courtois et al. (2021) proposed employing the adaptive LASSO to simplify the selection of tuning parameters. Note that while it is feasible to integrate multiple methods simultaneously within our framework, determining the optimal set of methods to use requires further investigation.

Hsieh et al. (2023) conducted a study that employs two signal detection methods simultaneously (Hsieh et al., 2023): sequence symmetry analysis (SSA; (Lai et al., 2017)) and tree-based scan statistics (TreeScan; (Kulldorff et al., 2013)). In their research, they examine two drugs using EHC data. Any ADR flagged by either SSA or TreeScan is treated as a signal. Consequently, their approach requires one to select appropriate thresholds for both methods, which can be challenging. In contrast, our approach simplifies this process by requiring only the selection of a threshold for the Borda count. How to extend their approach to accommodate an arbitrary number of methods and drugs is unclear.

Furthermore, research addresses the third phase, signal triage, by aiming to further refine the list of signals by filtering out known signals. In the same study by Hsieh et al. (2023), signals are automatically categorized into various groups, e.g., known ADRs, ADRs associated with indications and ADRs linked to patient characteristics. However, this study only considers two drugs. Subsequent research is need to explore how to extend this automated triage process to encompass more drugs. Thus far, we have not encountered any research proposing to enhance the post-market surveillance process with a verification step.

To facilitate the implementation of the approach described in this paper, we have developed an online tool accessible at https://borda.bips.eu.

## Data Availability

The data analyzed in this study is subject to the following licenses/restrictions: As we are not the owners of the data we are not legally entitled to grant access to the data of the German Pharmacoepidemiological Research Database. In accordance with German data protection regulations, access to the data is granted only to BIPS employees on the BIPS premises and in the context of approved research projects. Third parties may only access the data in cooperation with BIPS and after signing an agreement for guest researchers at BIPS. Requests to access these datasets should be directed to gepard@leibniz-bips.de.
