# Supplementary material for: A discovery and verification approach to pharmacovigilance using electronic healthcare data
Source: Front Pharmacol. 2024 Sep 4;15:1426323. doi: 10.3389/fphar.2024.1426323 (PMC11408326; doi:10.3389/fphar.2024.1426323)
Supplement: Supplementary file 1 [file DataSheet1.PDF]

Supplementary Material

A Discovery and Verification Approach to Pharmacovigilance using Electronic Healthcare Data

Louis Dijkstra[a], Tania Schink[a], Roland Linder[b], Markus Schwaninger[c], Iris Pigeot[a,d], Marvin N. Wright[a,d,e], and Ronja Foraita*[a]

[a]*Leibniz Institute for Prevention Research & Epidemiology – BIPS, Achterstraße 30, 28359 Bremen, Germany*
[b]*Techniker Krankenkasse – TK, Bramfelder Straße 140, 22305 Hamburg, Germany*
[c]*Institute for Experimental and Clinical Pharmacology and Toxicology, University of Lübeck, Ratzeburger Allee 160, 23562 Lübeck, Germany*
[d]*University of Bremen, Faculty of Mathematics and Computer Science, Bibliothekstraße 5, 28359 Bremen, Germany*
[e]*Section of Biostatistics, Department of Public Health, University of Copenhagen, Øster Farimagsgade 5, 1353 København K, Denmark*

In this supplementary material, we provide a formal definition of electronic healthcare data (EHC) and a description of the four signal detection methods used in the paper: the Bayesian confidence propagation neural network (BCPNN), the longitudinal gamma Poisson shrinker (LGPS), LASSO and random forests (RFs). Section S2 contains a formal definition of the Borda count, the Borda ranking and the relative Borda ranking for signal detection. The definition of a health outcome can be found in Section S3. In Section S4, we describe the German Pharmacoepidemiological Research Database (GePaRD) which is the data source in this work. We give a complete description of the study cohorts used in the signal detection and the verification phases in our case study in Section S5 and S7, respectively. The ICD-10-GM codes used for the signals are presented in Section S6 and Table S2. The covariates used as potential confounders are given in Section S8.

Contents

S1 Signal Detection Methods **2**
- S1.1 A formalization of electronic healthcare data 2
- S1.2 A formalization of a signal detection method 3
- S1.3 Bayesian confidence propagation neural network (BCPNN) 4
- S1.4 Longitudinal gamma Poisson shrinker 4
- S1.5 LASSO 5
- S1.6 Random forests 5

*Corresponding author. E-mail: foraita@leibniz-bips.de

S2 Borda count ranking **6**

S3 Definition health outcome **7**

S4 Data source (GePaRD) **7**

S5 Cohort signal detection study **7**

S6 The ICD-10-GM codes for health outcomes **8**

S7 Cohort verification study **8**

S8 Confounders included in the verification study **11**

S1 Signal Detection Methods

In this section, we define each of the four signal detection methods used in the paper: the longitudinal gamma Poisson shrinker (LGPS), the Bayesian confidence propagation neural network (BCPNN), LASSO and random forests (RF). We first provide a formal definition of electronic healthcare (EHC) data.

S1.1 A formalization of electronic healthcare data

EHC data contains for multiple patients 1) the drugs they were exposed to, and 2) the ADRs they experienced over time. We denote the number of drugs by m; the number of ADRs and the number of observed patients are denoted by n and N, respectively. The number of time points for which a patient was observed, can differ from patient to patient. We denote the total number of time points for the k-th patient by $T_k \geq 1$.

We represent the k-th patient's drug exposure to the i-th drug over time as a random binary T_k-dimensional vector:

$$\boldsymbol{X}_i^k = \left(X_i^k(1), X_i^k(2), \ldots, X_i^k(T_k)\right),$$

where $X_i^k(t) = 1$ if the k-th patient was exposed to the i-th drug at time point t, and 0 otherwise. Likewise, the occurrences of the j-th ADR are represented by the random T_k-dimensional binary vector

$$\boldsymbol{Y}_j^k = \left(Y_j^k(1), Y_j^k(2), \ldots, Y_j^k(T_k)\right),$$

where $Y_j^k(t) = 1$ if the k-th patient had the j-th ADR at time point t, and 0 otherwise. Since there are m drugs, we can represent all drug exposures for a patient k as a $(m \times T_k)$-dimensional random binary matrix, i.e.,

$$\boldsymbol{P}_{\text{drugs}}^k = \begin{bmatrix} \boldsymbol{X}_1^k \\ \boldsymbol{X}_2^k \\ \vdots \\ \boldsymbol{X}_m^k \end{bmatrix} = \begin{bmatrix} X_1^k(1) & X_1^k(2) & \ldots & X_1^k(T_k) \\ X_2^k(1) & X_2^k(2) & \ldots & X_2^k(T_k) \\ \vdots & \vdots & \ddots & \vdots \\ X_m^k(1) & X_m^k(2) & \ldots & X_m^k(T_k) \end{bmatrix},$$

where the rows represent the drugs and the T_k columns represent the time points. Likewise, we can represent the ADR history for patient k as an $(n \times T_k)$-dimensional binary matrix

$$\boldsymbol{P}^k_{\text{ADRs}} = \begin{bmatrix} \boldsymbol{Y}^k_1 \\ \boldsymbol{Y}^k_2 \\ \vdots \\ \boldsymbol{Y}^k_n \end{bmatrix} = \begin{bmatrix} Y^k_1(1) & Y^k_1(2) & \dots & Y^k_1(T_k) \\ Y^k_2(1) & Y^k_2(2) & \dots & Y^k_2(T_k) \\ \vdots & \vdots & \ddots & \vdots \\ Y^k_n(1) & Y^k_n(2) & \dots & Y^k_n(T_k) \end{bmatrix}.$$

Additional covariates associated with a patient, e.g., age and sex, can be represented by a V-dimensional vector $\boldsymbol{v}^k = \left(v^k_1, v^k_2, \dots, v^k_V\right)$ where $V \geq 0$ and $\boldsymbol{v}^k \in \mathbb{R}^V$.

The k-th patient is represented by both their drug exposure, ADR history and personal information, i.e.,

$$\boldsymbol{P}_k = \left\{ \boldsymbol{P}^k_{\text{drugs}}, \boldsymbol{P}^k_{\text{ADRs}}, \boldsymbol{v}^k \right\}.$$

An EHC data set is then a collection of N patients:

$$\mathbf{EHC} = \{\boldsymbol{P}_1, \boldsymbol{P}_2, \dots, \boldsymbol{P}_N\}.$$

Since $\boldsymbol{P}^k_{\text{drugs}} \in \{0,1\}^{m \times T_k}$, $\boldsymbol{P}^k_{\text{ADRs}} \in \{0,1\}^{n \times T_k}$ and $\boldsymbol{v}^k \in \mathbb{R}^V$, an EHC lies in the space $\mathscr{E} \in \{0,1\}^{T_+ mn} \times \mathbb{R}^{N \cdot V}$, where $T_+ = \sum_{k=1}^N T_k$ is the total number of observed time points in the EHC data set.

Observations are denoted by lower-case letters throughout: $\mathbf{ehc} = \{\boldsymbol{p}_k\}_{k=1}^N$ is a given EHC data set, where $\boldsymbol{p}_k = \{\boldsymbol{p}^k_{\text{drugs}}, \boldsymbol{p}^k_{\text{ADRs}}\}$ is the k-th patient. The rows of matrix $\boldsymbol{p}^k_{\text{drugs}}$ represent the observed drug exposures: $\boldsymbol{x}^k_i = (x^k_i(1), x^k_i(2), \dots, x^k_i(T_k))$ for $i = 1,2,\dots,m$. Similarly, the rows of matrix $\boldsymbol{p}^k_{\text{ADRs}}$ represent the observed ADRs: $\boldsymbol{y}^k_j = (y^k_j(1), y^k_j(2), \dots, y^k_j(T_k))$ for $j = 1,2,\dots,n$.

We assumes *patient independence* throughout, meaning that the joint probability density function of $\mathbf{EHC}$ can be factorized as

$$\mathbb{P}(\mathbf{EHC} = \mathbf{ehc}) = \prod_{k=1}^{N} \mathbb{P}(\boldsymbol{P} = \boldsymbol{p}_k),$$

where $\mathbb{P}(\boldsymbol{P})$ denotes the probability density function of a single patient.

S1.2 A formalization of a signal detection method

We define a signal detection method as a function $M : \mathscr{E} \to \mathbb{R}^{mn}$ that maps the EHC data to mn scores, one for each drug-ADR pair:

$$M(\mathbf{EHC}) = \{S_{ij} : i = 1,\dots,m \text{ and } j = 1,\dots,n\},$$

where S_{ij} is a real-valued random variable representing the score assigned to the drug-ADR pair (i,j). We denote an observation of S_{ij} by s_{ij}. The drug-ADR pairs are ranked on the basis of the $\{s_{ij}\}$ values. The rank for an individual drug-ADR pair (i,j) is denoted as $\text{rank}_{ij} \in \{1,2,\dots,mn\}$. We use the convention that the lower the rank, the stronger the signal, i.e., the pair with rank 1 is the strongest signal, and the pair with the highest rank is the weakest.

Table S1: Number of occurrences (#) for drug-ADR pair (i, j)

	# ADR j did occur	# ADR j did not occur	*total*
# drug i was prescribed	A_{ij}	B_{ij}	$A_{ij}+B_{ij}$
# drug i was not prescribed	C_{ij}	D_{ij}	$C_{ij}+D_{ij}$
total	$A_{ij}+C_{ij}$	$B_{ij}+D_{ij}$	$T_{+}=\sum_{k=1}^{N} T_k$

S1.3 Bayesian confidence propagation neural network (BCPNN)

The BCPNN was originally proposed for spontaneous reporting systems (SRSs; [Bate et al., 1998, Norén et al., 2006]). In order to apply this method to EHC data, one first needs to transform the EHC data to mn different 2×2 contingency tables, one for each drug-ADR pair in the data set [Zorych et al., 2011]. The BCPNN is then applied to the resulting tables, as if they were obtained from a SRS. See Table S1 for the 2×2 contingency table for drug-ADR pair (i, j). The counts A_{ij}, B_{ij}, C_{ij} and D_{ij} given in this table are defined as

$$\begin{aligned} A_{ij} &= \sum_{k=1}^{N}\sum_{t=1}^{T_k} X_i^k(t) Y_j^k(t), \quad B_{ij} = \sum_{k=1}^{N}\sum_{t=1}^{T_k} X_i^k(t)(1-Y_j^k(t)), \\ C_{ij} &= \sum_{k=1}^{N}\sum_{t=1}^{T_k} (1-X_i^k(t)) Y_j^k(t) \quad \text{and} \quad D_{ij} = \sum_{k=1}^{N}\sum_{t=1}^{T_k} (1-X_i^k(t))(1-Y_j^k(t)). \end{aligned} \tag{1}$$

In other words, A_{ij} is the total number of time points for which the patients were exposed to the i-th drug and experienced the j-th ADR; B_{ij} is the number of time points the patients were exposed but did not experience the ADR etc. The sum of these counts are the total number of observed time points, i.e., $A_{ij}+B_{ij}+C_{ij}+D_{ij} = \sum_{k=1}^{N} T_k$.

S1.4 Longitudinal gamma Poisson shrinker

Schuemie [2011] proposed the longitudinal gamma Poisson shrinker (LGPS) which is an adaptation of the GPS originally developed by DuMouchel for SRSs [DuMouchel, 1999]. Schuemie assumes that the number of time points a patient is exposed to the i-th drug and experiences the j-th ADR follows a Poisson distribution with mean μ_{ij} [Schuemie, 2011]. We can express this assumption as

$$A_{ij} \sim \text{Poisson}\left(\mu_{ij}\right),$$

where A_{ij} is given in eq. (1). Schuemie is interested in the quantity μ_{ij}/e_{ij}, where e_{ij} reflects the number of time points one would expect if the rate with which the j-th ADR occurs is the same if the patient is exposed or is not exposed to the i-th drug [Schuemie, 2011], i.e.,

$$e_{ij} = \left(a_{ij}+b_{ij}\right)\left(\frac{c_{ij}}{c_{ij}+d_{ij}}\right),$$

where a_{ij}, b_{ij}, c_{ij} and d_{ij} are the observed counts from Table S1. The sum $a_{ij}+b_{ij}$ is the total number of time points in which patients were exposed and $c_{ij}/(c_{ij}+d_{ij})$ reflects the rate with which the ADR occurs when a patient is not exposed. Schuemie and DuMouchel model the posterior distribution of the fraction μ_{ij}/e_{ij} as a mixture of Gamma distributions and use its mean as the score for the drug-ADR pair (i, j). See for more details the respective publications [Schuemie, 2011, DuMouchel, 1999].

S1.5 LASSO

Penalized regression in the form of the LASSO can be used for EHC data as well. This requires one to transform the EHC data, while maintaining their longitudinal nature. We introduce the random binary variables $\{W_i\}_{i=1}^m$, where W_i is 1 if a patient was ever exposed to the i-th drug, and 0 otherwise. Formally, we can write $W_i = \mathbb{1}\{\exists t \text{ such that } X_i(t) = 1\}$. In addition, we introduce the random binary variables $\{Z_{ij}\}_{i=1,\ldots,m}^{j=1,\ldots,n}$, one for each drug-ADR pair. The variable Z_{ij} is 1 if the following three conditions hold:

1. the patient was exposed to the i-th drug at least once, i.e., $W_i = 1$;
2. the patient experienced the j-th ADR at least once, i.e., there exists a t for which $Y_j(t) = 1$, and
3. the ADR occurred only while or after the patient was exposed:

$$\min\{\tau = 1,2,\ldots,T \text{ such that } X_i(t) = 1\} \leq \\ \min\{\tau = 1,2,\ldots,T \text{ such that } Y_j(t) = 1\}.$$

The variable Z_{ij} is 0 otherwise. The third condition reflects the fact that the drug can only be a cause of the ADR if the patient was exposed prior. We denote observed values of $\{W_i\}$ and $\{Z_{ij}\}$ with lower case letter, i.e., $\{w_i^k\}$ and $\{z_{ij}^k\}$, where $k = 1,2,\ldots,N$.

We are interested in estimating β_{i0}, $\beta_i = (\beta_{i1}, \beta_{i2}, \ldots, \beta_{in})$ and $\gamma_i = (\gamma_{i1}, \gamma_{i2}, \ldots, \gamma_{iV})$ in the penalized logistic regression problem

$$\left(\widehat{\beta}_{i0}, \widehat{\beta}_i, \widehat{\gamma}_i\right) = \underset{(\beta_{i0},\beta_i,\gamma_i)\in\mathbb{R}^{1+n+V}}{\arg\min} \Bigg\{ -\sum_{k=1}^{N} \Big[w_i^k \left(\beta_{i0} + \textstyle\sum_{j=1}^n \beta_{ij} z_{ij}^k + \sum_{l=1}^V \gamma_{il} v_l^k \right) \\ - \log\left(1 - \exp\left(\beta_{i0} + \textstyle\sum_{j=1}^n \beta_{ij} z_{ij}^k + \sum_{l=1}^V \gamma_{il} v_l^k\right)\right) \Big] + \lambda_i \|\beta_i\|_1 \Bigg\}.$$

The score assigned to the drug-ADR pair (i, j) is the estimated regression coefficient $\widehat{\beta}_{ij}$. Each tuning parameter λ_i is chosen based on cross-validation.

S1.6 Random forests

A random forest (RF [Breiman, 2001]) is an ensemble of decision trees, where each tree is based on a bootstrap sample of the original data, a process also referred to as bootstrap aggregating (bagging). Besides bagging, RFs introduce an additional source of randomness in that at each split the variable selected can only come from a random subset of variables.

Let us consider the random variables $\{W_i\}$ and $\{Z_{ij}\}$ introduced in the previous section, which denote whether the patients were exposed and whether the ADR of interest ocurred during or after the first drug exposure. We denote the observed values as $\boldsymbol{w}_i = \{w_i^k\}$ and $\boldsymbol{z}_{ij} = \{z_{ij}^k\}$. The patients' personal information is captured by $\boldsymbol{v} = \{\boldsymbol{v}^k\}_{k=1}^N$. When employing RFs for signal detection, one grows for each individual drug $i = 1,2,\ldots,m$ a forest with $b = 1,2,\ldots,B$ trees. Each tree is grown according to the following procedure [Breiman, 2001]:

1. Sample with replacement N patients. The resulting bootstrap sample is denoted by $\boldsymbol{w}_i^b$, $\boldsymbol{z}_{ij}^b$ and $\boldsymbol{v}_b$;

2. Repeat recursively for each terminal node the following three steps until a stopping condition has been met, e.g., the minimum node size reaches a predefined threshold;

   (a) Select $\widetilde{n} \leq n$ ADRs (variables) at random;

   (b) Pick the best split among the variables selected in the previous step based on the bootstrap sample $\boldsymbol{w}_i^b$, $\boldsymbol{z}_{ij}^b$ and $\boldsymbol{v}_b$, and

   (c) Split the selected node into two nodes.

3. Output the resulting tree T_b^i.

The result is a RF which is the collection of all grown trees, i.e., $F_i = \{T_b^i\}_{b=1}^B$. The score assigned to the drug-ADR pair (i, j) is the importance value of variable j given the RF F_i. There are many ways to conceptualize variable importance. In this work, we use the corrected version of the impurity measure [Nembrini et al., 2018]. We use the `R` package `ranger` for the analysis [Wright and Ziegler, 2017].

S2 Borda count ranking

Each signal detection method assigns a score for each drug-ADR pair on the basis of which a ranking of all pairs is created, see Section S1.2. Recall that we use the convention that the lower the ranking, the stronger the signal is thought to be.

Rather than using a single signal detection method, we propose in this work to use an ensemble of methods, in which the output of various methods are aggregated into a single score [Emerson, 2013]. The nature of the scores can differ between methods, e.g., the LASSO assigns penalized regression coefficients and the RF use variable importance values. Combining these scores themselves directly is, therefore, non-trivial. We propose to use the rankings rather than the scores and use the Borda count; a method to combine rankings with its origin in election mathematics [Emerson, 2013].

Suppose the ensemble consists of $q = 1, 2, \ldots, Q$ methods. The ranks assigned to drug-ADR pair (i, j) are denoted by $\{\text{rank}_{ij}^q\}_{q=1}^Q$, see Section S1.2. The ensemble method then assigns the Borda count as the *score* of drug-ADR pair (i, j), i.e.,

$$M_{\text{Borda}}(\mathbf{EHC}) = \left\{ S_{ij}^{\text{Borda}} = \sum_{q=1}^{Q} w_q \cdot \text{rank}_{ij}^q \text{ for } i = 1, 2, \ldots, m \text{ and } j = 1, 2, \ldots, n \right\}$$

where $\{w_q\}$ are positive weights (in our case, $w_q = 1$ for all q) and $S_{ij}^{\text{Borda}} \in \{Q, Q+1, \ldots, Qmn\}$ is the Borda count. Note that the minimal value of S_{ij}^{Borda} is Q, which corresponds to the case where the drug-ADR pair (i, j) is ranked first by all methods, and the maximal value is Qmn when all methods rank the pair last (the rank is mn). The weights w_q allows one to give different signal detection methods more or less weight. The *Borda count ranking* is based on the scores in $M_{\text{Borda}}(\mathbf{EHC})$. The Borda rank of drug-ADR pair (i, j) is denoted by $\text{rank}_{ij}^{\text{Borda}} \in \{1, 2, \ldots, mn\}$. One can also use the *relative Borda count ranking* which lies in the interval $[0, 1]$, i.e.,

$$\text{rank}_{ij}^{\text{rel. Borda}} = \frac{\text{rank}_{ij}^{\text{Borda}} - 1}{mn - 1}$$

for all $i = 1, 2, \ldots, m$ and $j = 1, 2, \ldots, n$.

S3 Definition health outcome

Based on medical or pharmacological considerations, the committee may consolidate multiple signals into a singular *health outcome*. Formally, we can denote a health outcome $\mathscr{H}$ as a subset of all drug-ADR pairs, i.e.,

$$\mathscr{H} = \mathscr{I} \times \mathscr{J} \qquad \text{where } \mathscr{I} \subseteq \{i = 1, 2, \ldots, m\} \text{ and } \mathscr{J} \subseteq \{j = 1, 2, \ldots, n\}, \tag{2}$$

where $\mathscr{I}$ and $\mathscr{J}$ represent the indices of drugs and ADRs, respectively. The pharmacoepidemiological study can be centered around a health outcome, rather than focusing on individual drugs and ADRs. This means that a patient is classified as a ‘case’ if exposed to any of the drugs in $\mathscr{I}$ and is considered to have experienced the reaction of interest if the ADR is in $\mathscr{J}$. The committee established various health outcomes during the signal triage phase of the case study, see Table S2.

S4 Data source (GePaRD)

We used GePaRD for the signal detection as well as for the verification phase of the case study. GePaRD is based on claims data from four statutory health insurance providers in Germany and currently includes information on approximately 25 million persons who have been insured with one of the participating providers since 2004 or later [Haug and Schink, 2021]. In addition to demographic data, GePaRD contains information on drug dispensations as well as outpatient (i.e., from general practitioners and specialists) and inpatient services and diagnoses. Per data year, there is information on approximately 20% of the living population and all geographical regions of Germany are represented. The suitability of GePaRD for pharmacoepidemiological research has been demonstrated by various types of pharmacoepidemiological studies investigating the safety of drugs [Haug and Schink, 2021].

In Germany, transferring health insurance data for scientific research without asking for informed consent is regulated by the Code of Social Law. All involved health insurance providers as well as the German Federal Office for Social Security and the Senator for Health, Women and Consumer Protection in Bremen as their responsible authorities approved this study according to the legal conditions. According to the Ethics Committee of the University of Bremen studies based on GePaRD are exempt from institutional review board review.

For more information, please visit the webpage: `https://www.bips-institut.de/en/research/research-infrastructures/gepard.html`

S5 Cohort signal detection study

The study cohort for the signal detection phase of our case study contained all persons who

1. had been insured with the health insurance Techniker Krankenkasse (TK) for at least 12 consecutive months between January 2015 and December 2016;
2. had at least one dispensation of rivaroxaban between 2015-04-02 and 2016-10-02, i.e., at least 90 days after cohort entry and before cohort exit, respectively, and
3. had no DOAC use within 12 months preceding cohort entry.

Persons were excluded from the study cohort if they switched to another DOAC. We use 4-digit ICD-10-GM codes for detecting potential signals.

S6 The ICD-10-GM codes for health outcomes

The ICD-10-GM codes used in the verification phase in our case study for the health outcomes can be found in Table S2. See Section S3 for the definition of a health outcome.

Table S2: The ADRs (expressed in ICD-10-GM codes) for the selected health outcomes

**Health outcome** ($\mathscr{H}$)	**ICD-10-GM**
Gastrointestinal bleeding	I98.3, K22.6, K22.8, K22.80, K22.81, K22.88, K25.0, K25.2, K25.4, K25.6, K26.0, K26.2, K26.4, K26.6, K27.0, K27.2, K27.4, K27.6, K28.0, K28.2,K 28.4, K28.6, K29.0, K31.8, K55.2, K55.3, K55.8, K57.0, K57.1, K57.2, K57.3, K57.4, K57.5, K57.8, K57.9, K62.5, K66.1, K92.0, K92.1, K92.2
Intracranial bleeding	I60.0, I60.1, I60.2, I603, I60.4, I60.5, I60.6, I60.7, I60.8, I60.9, I61.0, I61.1, I61.2, I61.3, I61.4, I61.5, I61.6, I61.8, I61.9, I62.0, I62.00, I62.01, I62.02, I62.09, I62.1, I62.9, S06.3, S06.4, S06.5, S06.6
Acute liver injury	K71.0, K71.1, K71.2, K71.6, K71.7, K71.8, K71.9, K72.0, K72.9, K75.2, K75.3, K75.8, K75.9, K76.2
Acute cystitis	N30.0
Epilepsy	G4008, G4009, G401, G402, G403, G404, G405, G406, G407, G408, G409, R568
Sepsis	A02.1, A20.7, A22.7, A26.7, A32.7, A39.2, A39.3, A39.4, A40.0, A40.1, A40.2, A40.3, A40.8, A40.9, A41.0, A41.1, A41.2, A41.3, A41.4, A41.5, A41.8, A41.9, A42.7, B37.7

S7 Cohort verification study

The study cohort for the verification phase of our case study included all persons from January 2011 to December 2017 who

1. had at least one dispensation of RVX or PPC during the study period;
2. had been continuously insured for at least 12 months before cohort entry;
3. had no dispensing of RVX or PPC in the 12 months before cohort entry ('new user');
4. had at least one diagnosis of atrial fibrillation within 12 months before cohort entry, and
5. had no diagnosis of cancer any time before cohort entry and were not pregnant at cohort entry.

Exposure at index date was defined as a supply overlapping the index date, whereas supply was estimated as the number of defined daily doses of the last dispensing before the index day. Only hospital diagnoses were considered for the events acute liver injury, intracranial bleeding,

gastrointestinal bleeding and sepsis. For other ICD codes, outpatient diagnoses were also considered, which are classified as 'certain'. The index date was set to the respective admission date and patients with a respective diagnosis 180 days before cohort entry were excluded. For cystitis, both outpatient and hospital diagnoses were taken into account. The index date was set to the date of diagnosis or the admission date, and all patients with a respective diagnosis 365 days before cohort entry were excluded. The study cohort of the verification study was based on 97,400 new users of PPC and 71,917 users of RVX. The median age at cohort entry was 73 (IQR: 65, 79) for rivaroxaban and 75 (IQR: 69, 81) for PPC new users. The proportion of women was 47% (RVX) and 48% (PPC). We observed 5,053 cases of acute cystitis, 322 cases of acute liver injury, 3,504 cases of sepsis, 6,705 cases of gastrointestinal bleeding and 2,974 cases of intracranial bleeding. Baseline characteristics including relevant medical history and dispensed medication at cohort entry are presented in Table S2.

Table S3 describes some main characteristics of cases and controls for each health outcome.

Table S3: Characteristics of cases and matched controls at cohort entry

**Characteristics**	**Cases** *n* (%)	**Controls** *n* (%)
**Gastrointestinal bleeding**	6,705	67,019
Females	3,531 (53%)	35,296 (53%)
Age: *median (IQR)*	80.0 (74.0, 85.0)	80.0 (74.0, 85.0)
Medical history		
Acute renal failure	54 (0.8%)	189 (0.3%)
Heart failure	4,243 (63%)	34,330 (51%)
Hypertension	6,564 (98%)	64,057 (96%)
Moderate/severe chronic kidney disease	1,740 (26%)	10,862 (16%)
Liver disease	2,343 (35%)	18,931 (28%)
Medication use		
Antithrombotics	1,534 (23%)	11,275 (17%)
CYP-/P-glycoprotein inducers	30 (0.4%)	233 (0.3%)
**Intracranial bleeding**	2,974	29,720
Females 1,497	(50%)	14,970 (50%)
Age: *median (IQR)*	80.0 (74.0, 85.0)	80.0 (74.0, 85.0)
Alcohol and drug abuse	186 (6.3%)	1,235 (4.2%)
Medical history		
Brain disease	58 (2.0%)	244 (0.8%)
Ischemic stroke	628 (21%)	4,699 (16%)
Transient ischemic attack	168 (5.6%)	1,548 (5.2%)
Moderate/severe chronic kidney disease	584 (20%)	4,764 (16%)
Medication use		
Antithrombotics	553 (19%)	5,099 (17%)
CYP-/P-glycoprotein inducers	14 (0.5%)	125 (0.4%)
Systemic corticosteroids	605 (20%)	5,479 (18%)
Pethidine, Tramadol	320 (11%)	3,299 (11%)
**Acute cystitis**	29,698	296,532
Females	18,885 (64%)	188,535 (64%)

Table S3: Characteristics of cases and matched controls at cohort entry

**Characteristics**	**Cases**<br>*n* (%)	**Controls**<br>*n* (%)
Age: *median (IQR)*	77.0 (71.0, 83.0)	77.0 (71.0, 83.0)
Alcohol and drug abuse	1,405 (4.7%)	12,323 (4.2%)
Medical history		
Genital abnormalities	4,756 (16%)	38,456 (13%)
Other disorders related to cystitis	7,445 (25%)	68,339 (23%)
Prostatic disease	6,825 (23%)	59,606 (20%)
Urinary disorders	8,986 (30%)	59,716 (20%)
**Acute liver injury**	322	3,220
Females	184 (57%)	1,840 (57%)
Age: *median (IQR)*	75.0 (69.0, 80.0)	75.0 (69.0, 80.0)
Alcohol and drug abuse	31 (9.6%)	149 (4.6%)
Medical history		
Cardiovascular disease	203 (63%)	1,957 (61%)
Diabetes mellitus	146 (45%)	1,029 (32%)
Chronic Hepatitis	4 (1.2%)	18 (0.6%)
Other liver disease	139 (43%)	919 (29%)
Medication use		
Antibiotics[a]	9 (2.8%)	46 (1.4%)
CYP-/P-glycoprotein inducers	3 (0.9%)	17 (0.5%)
NSAIDs	256 (80%)	2,619 (81%)
**Epilepsy**	3,070	30,676
Females	1,618 (53%)	16,175 (53%)
Age: *median (IQR)*	78 (72, 84)	78 (72, 84)
Alcohol and drug abuse	247 (8.0%)	1,317 (4.3%)
Medical history		
Cardiovascular disease	2,125 (69%)	19,832 (65%)
Brain disease	68 (2.2%)	247 (0.8%)
Dementia	1,320 (43.0%)	10,978 (35.8%)
Ischemic stroke	1,103 (35.9%)	4,157 (13.6%)
Transient ischemic attack	262 (8.5%)	1,379 (4.5%)
Medication use		
Antibiotics[a]	105 (3.4%)	517 (1.7%)
Antiepileptic drugs[a]	459 (15%)	359 (1.2%)
Antipsychotics/antidepressants[a]	574 (19%)	1,993 (6.5%)
**Sepsis**	3,504	35,022
Females	1,529 (44%)	15,286 (44%)
Age: *median (IQR)*	79.0 (73.0, 84.0)	79.0 (73.0, 84.0)
Alcohol and drug abuse	264 (7.5%)	1,506 (4.3%)
Medical history		
Anemia	1,628 (46%)	10,577 (30%)

Table S3: Characteristics of cases and matched controls at cohort entry

Characteristics	Cases *n* (%)	Controls *n* (%)
Heart failure	2,368 (68%)	17,228 (49%)
Hypertension	3,445 (98%)	33,370 (95%)
Malnutrition	117 (3.3%)	376 (1.1%)
Moderate/severe chronic kidney disease	1,146 (33%)	5,368 (15%)
Medication use		
Anti-infective drugs[a]	261 (7.4%)2	532 (1.5%)2
CYP-/P-glycoprotein inducers[a]	23 (0.7%)2	123 (0.4%)2
Immunosuppressants	203 (5.8%)	777 (2.2%)

[a] Supply was overlapping the index

S8 Confounders included in the verification study

See for a detailed list of all confounders included in the verification study Table S4 at the end of this document. Details on epilepsy can be found in Platzbecker et al. [2023].

References

A. Bate, M. Lindquist, I. R. Edwards, S. Olsson, R. Orre, A. Lansner, and R. M. D. Freitas. A Bayesian neural network method for adverse drug reaction signal generation. *European Journal of Clinical Pharmacology*, 54(4):315–321, 1998. URL https://doi.org/10.1007%2Fs002280050466.

L. Breiman. Random forests. *Machine learning*, 45:5–32, 2001. URL https://doi.org/10.1023/A:1010933404324.

W. DuMouchel. Bayesian data mining in large frequency tables, with an application to the FDA spontaneous reporting system. *The American Statistician*, 53(3):177, 1999. URL https://doi.org/10.2307%2F2686093.

P. Emerson. The original Borda count and partial voting. *Social Choice and Welfare*, 40(2): 353–358, 2013. ISSN 01761714. doi: 10.1007/s00355-011-0603-9.

U. Haug and T. Schink. German pharmacoepidemiological research database (GePaRD). In *Databases for Pharmacoepidemiological Research*, pages 119–124. Springer International Publishing, 2021. URL https://doi.org/10.1007%2F978-3-030-51455-6_8.

S. Nembrini, I. R. König, and M. N. Wright. The revival of the Gini importance? *Bioinformatics*, 34(21):3711–3718, 2018. URL https://doi.org/10.1093%2Fbioinformatics%2Fbty373.

G. N. Norén, A. Bate, R. Orre, and I. R. Edwards. Extending the methods used to screen the WHO drug safety database towards analysis of complex associations and improved accuracy for rare events. *Statistics in Medicine*, 25(21):3740–3757, 2006. URL https://doi.org/10.1002%2Fsim.2473.

K. Platzbecker, H. Müller-Fielitz, R. Foraita, M. J. Koepp, A. Voss, R. Pflock, R. Linder, I. Pigeot, T. Schink, and M. Schwaninger. In atrial fibrillation epilepsy risk differs between oral anticoagulants: Active comparator, nested case-control study. *Europace*, 25(5), 2023. doi: 10.1093/europace/euad087. URL `https://doi.org/10.1093/europace/Feuad087`.

M. J. Schuemie. Methods for drug safety signal detection in longitudinal observational databases: LGPS and LEOPARD. *Pharmacoepidemiology and Drug Safety*, 20(3):292–299, 2011. URL `https://doi.org/10.1002%2Fpds.2051`.

M. N. Wright and A. Ziegler. ranger: A fast implementation of random forests for high dimensional data in C++ and R. *Journal Statistical Software*, 2017. URL `https://doi.org/10.18637/jss.v077.i01`.

I. Zorych, D. Madigan, P. Ryan, and A. Bate. Disproportionality methods for pharmacovigilance in longitudinal observational databases. *Statistical Methods in Medical Research*, 22 (1):39–56, 2011. URL `https://doi.org/10.1177%2F0962280211403602`.

Table S4. Detailed description of confounders: ICD-10-GM and ATC codes used to define the respective confounder variables.

ACUTE CYSTITIS		
**Diagnoses**	**Codes (ICD-10-GM if not further specified)**	**Measurement period**
**Neurogenic bladder dysfunction**	**N31.x** Neuromuscular dysfunction of bladder, not elsewhere classified	Any time prior cohort entry
**Circumcision**	**Z41.2** Circumcision as a routine measure or for ritual reasons	Half a year prior cohort entry
**Urinary dysfunction**	**R33** Retention of urine<br>**R39.1** Other difficulties with micturition	Half a year prior cohort entry
**Genital prolapse**	**N81.x** Female genital prolapse<br>**N83.4** Prolapse and hernia of ovary and fallopian tube<br>**N99.0** Kidney failure after medical measures<br>**N99.3** Prolapse of the vaginal stump after hysterectomy	Any time prior cohort entry
**Sexual dysfunction**	**F52.7** Excessive sexual drive	Any time prior cohort entry
**Intrauterine device**	**Z30.1** Insertion of (intrauterine) contraceptive device<br>**Z30.5** Surveillance of (intrauterine) contraceptive device	Any time prior cohort entry
**Prostatic hypertrophy**	**N40** Hyperplasia of prostate	Any time prior cohort entry
**Prosthetic devices**	**T83.5** Infection and inflammatory reaction due to prosthetic device, implant and graft in urinary system<br>**T83.6** Infection and inflammatory reaction due to prosthetic device, implant and graft in genital tract	Any time prior cohort entry
**Urinary incontinence**	**N39.4x** Other specified urinary incontinence<br>**R32** Unspecified urinary incontinence	Any time prior cohort entry
**Urinary tract obstruction**	**N13.x** Obstructive and reflux uropathy	1-year prior cohort entry
**Vulvovaginal atrophy**	**N90.5** Atrophy of vulva	Any time prior cohort entry
**Medications**	**Codes (ATC)**	**Measurement period**
**Immuno-suppressants**	**L04A** IMMUNOSUPPRESSANTS	Half a year prior index date
ACUTE LIVER INJURY		
**Diagnoses**	**Codes (ICD-10-GM if not further specified)**	**Measurement period**
**Alcohol and drug abuse**	**E52** Niacin deficiency<br>**G31.2** Degeneration of nervous system due to alcohol<br>**G62.1** Alcoholic polyneuropathy<br>**G72.1** Alcoholic myopathy<br>**I42.6** Alcoholic cardiomyopathy<br>**K29.2** Alcoholic gastritis<br>**K70.x** Alcoholic liver disease	Any time prior cohort entry
	**O35.4** Maternal care for (suspected) damage to fetus from alcohol<br>**P04.3** Fetus and newborn affected by maternal use of alcohol<br>**Q86.0** Fetal alcohol syndrome (dysmorphic)<br>**Z50.2** Care involving use of rehabilitation procedures: Alcohol rehabilitation	1 year prior cohort entry
	**E24.4** Alcohol-induced pseudo-Cushing syndrome<br>**K85.2** Alcohol-induced acute pancreatitis<br>**K86.0** Alcohol-induced chronic pancreatitis<br>**R78.0** Finding of alcohol in blood<br>**T51.0** Toxic effect of alcohol: Ethanol<br>**T51.9** Toxic effect of alcohol: Alcohol, unspecified<br>**Z72.0** Alcohol use	Half a year prior cohort entry
**Liver disease**	**K71.x** Toxic liver disease<br>**K72.x** Hepatic failure, not elsewhere classified<br>**K73.x** Chronic hepatitis, not elsewhere classified<br>**K74.x** Fibrosis and cirrhosis of liver<br>**K75.x** Other inflammatory liver diseases<br>**K76.x** Other disease of liver<br>**K77.x** Liver disorders in diseases classified elsewhere<br>**Z94.4** Liver transplant status	Any time prior cohort entry
	**K75.8** Other specified inflammatory liver diseases	Half a year prior cohort entry
**Hepatitis**	**B18.x** Chronic viral hepatitis	Any time prior cohort entry
	**B15.x** Acute hepatitis A<br>**B16.x** Acute hepatitis B<br>**B17.x** Other acute viral hepatitis<br>**B19.x** Unspecified viral hepatitis	Half a year prior cohort entry
**Metabolic disorder**	**E88.0** Disorders of plasma-protein metabolism, not elsewhere classified	Any time prior cohort entry
**Primary sclerosing cholangitis**	**K83.x** Other diseases of biliary tract	Half a year prior cohort entry
**Toxic ingestions**	**T64** Toxic effect of aflatoxin and other mycotoxin food contaminants	Half a year prior cohort entry
**Medications**	**Codes (ATC)**	**Measurement period**
**Antibiotics**	**J01** ANTIBACTERIALS FOR SYSTEMIC USE	Half a year prior cohort entry

	**J01A** TETRACYCLINES<br>**J01FA10** Azithromycin<br>**R05GB07** Erythromycin, combinations	
**Antihypertensives**	**C02** Antihypertensives<br>**C02AB01** Methyldopa (levorotatory)<br>**C02AB02** Methyldopa (racemic)<br>**C02LB01** Methyldopa (levorotatory) and diuretics	Half a year prior cohort entry
**Antiinflammatory and antirheumatic procuducts, non-steroids**	**M01A** ANTIINFLAMMATORY AND ANTIRHEUMATIC PRODUCTS, NON-STEROIDS	Half a year prior cohort entry
**Diclofenac**	**M01AB05** Diclofenac<br>**M01AB55** Diclofenac, combinations<br>**M02AA15** Diclofenac<br>**N02AJ05** Diclofenac, combinations<br>**S01BC03** Diclofenac<br>**S01CC01** Diclofenac and antiinfectives	Half a year prior cohort entry
**Paracetamol**	**N02AJ01** Dihydrocodeine and paracetamol<br>**N02AJ06** Oxycodone and paracetamol<br>**N02AJ17** Codeine and paracetamol<br>**N02BE01** Paracetamol<br>**N02BE51** Paracetamol, combinations excl. psycholeptics<br>**N02BE61** Paracetamol, combinations with coffeine<br>**N02BE71** Paracetamol, combinations with psycholeptics<br>**N02CX63** Paracetamol, combination<br>**N02CX69** Metoclopramid and paracetamol	
**CYP-/P-glycoprotein inhibitors**	**J01FA01** Erythromycin<br>**J01FA09** Clarithromycin<br>**J01FA15** Telithromycin<br>**J02AC02** Itraconazol<br>**J05AE09** Tipranavir<br>**S01AA17** Erythromycin	Half a year prior cohort entry
**CYP-/P-glycoprotein inducer**	**J04AM05** Rifampicin, pyrazinamide and isoniazid<br>**J04AM06** Rifampicin, pyrazinamide, ethambutol and isoniazid	Half a year prior cohort entry
**Ophthalmologicals**	**S01AA26** Azithromycin	Half a year prior cohort entry
**Drugs for treatment of tuberculosis**	**J04AC01** Isoniazid<br>**J04AC51** Isoniazid, combinations<br>**J04AK01** Pyrazinamide<br>**J04AM** Combinations of drugs for treatment of tuberculosis	Half a year prior cohort entry
**Virustatics**	**J05AE** Protease inhibitors<br>**J05AX09** Maraviroc<br>**J05AE09** Tipranavir	Half a year prior cohort entry
**Other drugs**	**J02AC02** Itraconazole<br>**L01XE11** Pazopanib<br>**N02AJ13** Tramadol und Paracetamol<br>**N02BA** Salicylic acid and derivatives	Half a year prior cohort entry
**EPILEPSY**	(see Platzberger et al., 2023)	
**GASTROINTESTINAL AND INTRACRANIAL BLEEDING**		
**Diagnoses**	**Codes (ICD-10-GM if not further specified)**	**Measurement period**
**Acute hemorrhagic conjunctivitis**	**B30.3** Acute epidemic hemorrhagic conjunctivitis (enteroviral)<br>**S05.x** Injury of eye and orbit	Half a year prior cohort entry
**Alcohol and drug abuse**	**F10.x** Mental and behavioral disorders due to alcohol	Half a year prior cohort entry
	**K70.x** Alcoholic liver disease	Any time prior cohort entry
**Liver disease**	**K72.x** Hepatic failure, not elsewhere classified<br>**K74.x** Fibrosis and cirrhosis of liver	Any time prior cohort entry
**Diseases of the gastrointestinal tract**	**K21.x** Gastro-oesophageal reflux disease<br>**K29.0** Acute haemorrhagic gastritis<br>**K29.1** Other acute gastritis<br>**K29.2** Alcoholic gastritis<br>**K29.6** Other gastritis<br>**K29.7** Gastritis, unspecified<br>**K29.8** Duodenitis<br>**K29.9** Gastroduodenitis, unspecified	Half a year prior cohort entry
**Chronic diseases of the genitourinary system**	**N70.1** Chronic salpingitis and oophoritis<br>**N80.x** Endometriosis	Any time prior cohort entry
**Acute diseases of the genitourinary system**	**N70.0** Acute salpingitis and oophoritis<br>**N70.9** Salpingitis and oophoritis, unspecified	Half a year prior cohort entry
**Chronic diseases of the gastrointestinal tract**	**K29.3** Chronic superficial gastritis<br>**K29.4** Chronic atrophic gastritis<br>**K29.5** Chronic gastritis, unspecified	Any time prior cohort entry

**Bleedings**	**D57.0** Sickle-cell anaemia with crisis<br>**D57.1** Sickle-cell anaemia without crisis<br>**D65.x** Disseminated intravascular coagulation<br>**D66** Hereditary factor VIII deficiency<br>**D67** Hereditary factor IX deficiency<br>**D68.1** Hereditary factor XI deficiency<br>**D68.2x** Hereditary deficiency of other clotting factors<br>**D68.4** Acquired coagulation factor deficiency<br>**D68.5** Primary thrombophilia<br>**D68.6** Other thrombophilia<br>**D68.8** Other specified coagulation defects<br>**D68.9** Coagulation defect, unspecified<br>**D69.0** Allergic purpura<br>**D69.1** Qualitative platelet defects<br>**D69.2** Other nonthrombocytopenic purpura<br>**D69.3** Idiopathic thrombocytopenic purpura<br>**D69.8** Other specified haemorrhagic conditions<br>**D69.9** Haemorrhagic condition, unspecified	Any time prior cohort entry
	**D68.3x** Haemorrhagic disorder due to circulating anticoagulants<br>**D69.4x** Other primary thrombocytopenia<br>**D69.5x** Secondary thrombocytopenia<br>**D69.6x** Thrombocytopenia, unspecified	1-year prior cohort entry
	**Z98.x** Invasive interventions: Other postsurgical states	Half a year prior cohort entry
**Burn**	**T27.x** Burn and corrosion of respiratory tract	Half a year prior cohort entry
**Rare disorders**	**M30.0** Panarteritis nodosa	Any time prior cohort entry
**Metabolic disorders**	**E84.x** Cystic fibrosis<br>**E85.x** Amyloidosis	Any time prior cohort entry
**Congenital malformations**	**Q51.x** Congenital malformations of uterus and cervix	Any time prior cohort entry
**History of cystitis**	**N30.x** Cystitis<br>**N34.x** Urethritis	1-year prior cohort entry
**Hypertension**	**I10.x** Essential (primary) hypertension	Any time prior cohort entry
**Hypercalciuria**	**E83.5x** Disorders of calcium metabolism	Any time prior cohort entry
**Pulmonary embolism**	**I26.x** Pulmonary embolism	1-year prior cohort entry
**Malaria**	**B53.0** Plasmodium ovale malaria	Any time prior cohort entry
	**B50.x** Plasmodium falciparum malaria<br>**B51.x** Plasmodium vivax malaria<br>**B52.x** Plasmodium malariae malaria<br>**B53.1** Malaria due to simian plasmodia<br>**B53.8** Other parasitologically confirmed malaria, not elsewhere classified<br>**B54** Unspecified malaria	Half a year prior cohort entry
**Disease of pericardium**	**I31.9** Disease of pericardium, unspecified<br>**I78.0** Hereditary haemorrhagic telangiectasia	Any time prior cohort entry
**Other injuries**	**H43.8** Other disorders of vitreous body<br>**S02.1** Fracture of base of skull<br>**S09.2** Traumatic rupture of ear drum<br>**S26.0** Injury of heart with haemopericardium<br>**T70.2** Other and unspecified effects of high altitude	Half a year prior cohort entry
**Parasites**	**B66.4** Paragonimiasis	Half a year prior cohort entry
**Pulmonary artery-venous malformation**	**I28.0** Arteriovenous fistula of pulmonary vessels<br>**I28.1** Aneurysm of pulmonary artery	1-year prior cohort entry
**Pneumonia**	**J13** Pneumonia due to Streptococcus pneumoniae<br>**J14** Pneumonia due to Haemophilus influenzae<br>**J15.x** Bacterial pneumonia, not elsewhere classified<br>**J16.x** Pneumonia due to other infectious organisms, not elsewhere classified<br>**J17.x** Pneumonia in diseases classified elsewhere<br>**J18.x** Pneumonia, organism unspecified	Half a year prior cohort entry
**Tubal pregnancy**	**O.001** Tubal pregnancy	Half a year prior cohort entry
**Prostatitis**	**N41.1** Chronic prostatitis	Any time prior cohort entry
	**N41.x** Inflammatory diseases of prostate	Half a year prior cohort entry
**Diseases of the respiratory system**	**J47** Bronchiectasis	Any time prior cohort entry
	**J20.x** Acute bronchitis<br>**J85.x** Abscess of lung and mediastinum	Half a year prior cohort entry
**Injury of urinary and pelvic organs**	**S37.x** Injury of urinary and pelvic organs	1-year prior cohort entry
**Systemic diseases of connective tissue**	**M31.3** Thrombotic microangiopathy<br>**M31.7** Microscopic polyangiitis	Any time prior cohort entry
**Sepsis**	**A40.x** Streptococcal sepsis<br>**A41.x** Other sepsis	Half a year prior cohort entry
**Systemic connective tissue disorders**	**M32.x** Systemic lupus erythematosus	Any time prior cohort entry

**Urolithiasis**	**N20.x** Calculus of kidney and ureter	Half a year prior cohort entry
**Tuberculosis**	**A18.1** Tuberculosis of genitourinary system	Any time prior cohort entry
	**A15.x** Respiratory tuberculosis, bacteriologically and histologically confirmed<br>**A16.x** Respiratory tuberculosis, not confirmed bacteriologically or histologically	1-year prior cohort entry
**Injuries of the thorax**	**S27.0** Traumatic pneumothorax<br>**S27.1** Traumatic haemothorax<br>**S27.2** Traumatic haemopneumothorax<br>**S27.3x** Other injuries of lung	Half a year prior cohort entry
**Tumor**	**D25.x** Leiomyoma of uterus	Any time prior cohort entry
**Medications**	**Codes (ATC)**	**Measurement period**
**Antithrombotic agents**	**B01** ANTITHROMBOTIC AGENTS<br>**B01AB** Heparin group<br>**B01AC** Platelet aggregation inhibitors excl. heparin<br>**B01AD** Enzymes<br>**B01AE01** Desirudin<br>**B01AE02** Lepirudin<br>**B01AE03** Argatroban<br>**B01AE06** Bivalirudin<br>**B01AX** Other antithrombotic agents<br>**B01AY** Enzymes for topical use	Half a year prior index date
**Anti-dementia drugs**	**N06DA53** Donepezil, memantine and Ginkgo biloba leaf dry extract<br>**N06DP01** Ginkgo biloba leaf dry extract<br>**N06DX02** Ginkgo folium	Half a year prior index date
**P-glycoprotein inhibitors**	**C10BX06** Atorvastatin, acetylsalicylic acid and ramipril<br>**C10BX08** Atorvastatin and acetylsalicylic acid<br>**C10BX12** Atorvastatin, acetylsalicylic acid and perindopril	Half a year prior index date
**Salicylic acid and derivatives**	**A01AD05** Acetylsalicylic acid<br>**C10BX01** Simvastatin and acetylsalicylic acid<br>**C10BX02** Pravastatin and acetylsalicylic acid<br>**C10BX04** Simvastatin, acetylsalicylic acid and ramipril<br>**C10BX05** Rosuvastatin and acetylsalicylic acid<br>**C10BX06** Atorvastatin, acetylsalicylic acid and ramipril<br>**C10BX08** Atorvastatin and acetylsalicylic acid<br>**C10BX12** Atorvastatin, acetylsalicylic acid and perindopril<br>**M01BA03** Acetylsalicylic acid and corticosteroids<br>**N02AJ02** Dihydrocodeine and acetylsalicylic acid<br>**N02AJ07** Codeine and acetylsalicylic acid<br>**N02AJ18** Oxycodone and acetylsalicylic acid<br>**R05XA02** Acetylsalicylic acid, combinations<br>**R05XA22** Acetylsalicylic acid and pseudoephedrin	Half a year prior index date
**Vasoprotectives**	**C05AX08** Heparin for topical use<br>**C05BA** Heparins or heparinoids for topical use	Half a year prior index date
**SEPSIS**		
**Diagnoses**	**Codes (ICD-10-GM if not further specified)**	**Measurement period**
**Alcohol and drug abuse**	**K70.x** Alcoholic liver disease	Any time prior cohort entry
**Liver disease**	**K70.x** Alcoholic liver disease<br>**K71.x** Toxic liver disease<br>**K72.x** Hepatic failure, not elsewhere classified<br>**K73.x** Chronic hepatitis, not elsewhere classified<br>**K74.x** Fibrosis and cirrhosis of liver<br>**K75.x** Other inflammatory liver diseases<br>**K76.x** Other disease of liver<br>**K77.x** Liver disorders in diseases classified elsewhere<br>**Z94.4** Liver transplant status	Half a year prior cohort entry
	**K75.8** Other specified inflammatory liver diseases	Half a year prior cohort entry
**Acute renal failure**	**K76.7** Hepatorenal syndrome	1 year prior cohort entry
**Chronic kidney disease**	**N18.1** Chronic kidney disease, stage 1<br>**N18.2** Chronic kidney disease, stage 2<br>**N18.3** Chronic kidney disease, stage 3<br>**N18.4** Chronic kidney disease, stage 4<br>**N18.5** Chronic kidney disease, stage 5<br>**N18.8x** Other chronic kidney disease, stage unspecified<br>**N18.9** Chronic kidney disease, unspecified<br>**N19** Unspecified kidney failure<br>**Z49.x** Care involving dialysis<br>**Z99.2** Dependence on renal dialysis	Any time prior cohort entry
**Chronic obstructive pulmonary disease**	**J43.x** Emphysema<br>**J44.x** Other chronic obstructive pulmonary disease	Any time prior cohort entry
**Hepatitis**	**B18.x** Chronic viral hepatitis	Any time prior cohort entry
	**B19.x** Unspecified viral hepatitis	Half a year prior cohort entry
**HIV**	**B20** Human immunodeficiency virus [HIV] disease resulting in infectious and parasitic diseases	Any time prior cohort entry

	**B21** Human immunodeficiency virus [HIV] disease resulting in malignant neoplasms<br>**B22** Human immunodeficiency virus [HIV] disease resulting in other specified diseases<br>**B23** Human immunodeficiency virus [HIV] disease resulting in other conditions<br>**B23.0** Acute HIV infection syndrome<br>**B23.8** HIV disease resulting in other specified conditions<br>**B24** Unspecified human immunodeficiency virus [HIV] disease<br>**U60** Clinical categories of HIV disease<br>**U61** Number of T helper cells in HIV disease<br>**Z21** Asymptomatic human immunodeficiency virus [HIV] infection status	
**Immunodeficiency**	**D80** Immunodeficiency with predominantly antibody defects<br>**D81** Combined immunodeficiencies<br>**D82** Immunodeficiency associated with other major defects<br>**D83** Common variable immunodeficiency<br>**D84** Other immunodeficiencies<br>**D86** Sarcoidosis<br>**D89** Other disorders involving the immune mechanism, not elsewhere classified<br>**D90** Immunocompromised after radiotherapy, chemotherapy and other immunosuppressive measures	Any time prior cohort entry
**Malnutrition**	**E40** Kwashiorkor<br>**E41** Nutritional marasmus<br>**E42** Marasmic kwashiorkor<br>**E43** Unspecified severe protein-energy malnutrition<br>**E44.x** Protein-energy malnutrition of moderate and mild degree<br>**E45** Retarded development following protein-energy malnutrition<br>**E46** Unspecified protein-energy malnutrition	Any time prior cohort entry
**Prosthetic device(s)**	**T82** Complications of cardiac and vascular prosthetic devices, implants and grafts<br>**T83** Complications of genitourinary prosthetic devices, implants and grafts<br>**T84.0x** Mechanical complication of internal joint prosthesis<br>**T84.1x** Mechanical complication of internal fixation device of bones of limb<br>**T84.2x** Mechanical complication of internal fixation device of other bones<br>**T84.3** Mechanical complication of other bone devices, implants and grafts<br>**T84.4** Mechanical complication of other internal orthopaedic devices, implants and grafts<br>**T84.5** Infection and inflammatory reaction due to internal joint prosthesis<br>**Z95.x** Presence of cardiac and vascular implants and grafts	Any time prior cohort entry
**Medications**	**Codes (ATC)**	**Measurement period**
**Anti- infectives**	**J01** ANTIBACTERIALS FOR SYSTEMIC USE<br>**J02A** ANTIMYCOTICS FOR SYSTEMIC USE<br>**J04AB02** Rifampicin<br>**J04AB04** Rifabutin<br>**J04AC01** Isoniazid<br>**J04AC51** Isoniazid, combination<br>**J04AK01** Pyrazinamid<br>**J04AM01** Streptomycin and isoniazid<br>**J04AM02** Rifampicin and isoniazid<br>**J04AM03** Ethambutol and isoniazid<br>**J04AM04** Thioacetazon and isoniazid<br>**J04AM05** Rifampicin, pyrazinamid and isoniazid<br>**J04AM06** Rifampicin, pyrazinamide, ethambutol and isoniazid<br>**J04AM07** Rifampicin, ethambutol and isoniazid<br>**J04AM08** Isoniazid, sulfamethoxazole, trimethoprim and pyridoxine<br>**J05AB01** Aciclovir<br>**J05AB06** Ganciclovir<br>**J05AB11** Valaciclovir<br>**J05AB12** Cidofovir<br>**J05AC04** Amantadin<br>**J05AD01** Foscarnet<br>**J05AE** Protease inhibitors<br>**J05AX09** Maraviroc	Half a year prior index date
**Immuno-suppressants**	**L04A** IMMUNOSUPPRESSANTS	Half a year prior index date
